# Antidiarrheal activity of crude methanolic root extract of *Idigofera spicata* Forssk.(Fabaceae)

**DOI:** 10.1186/s12906-016-1252-4

**Published:** 2016-08-05

**Authors:** Eshetie Melese Birru, Assefa Belay Asrie, Getnet Mequanint Adinew, Asegedech Tsegaw

**Affiliations:** Department of Pharmacology, College of Medicine and Health Sciences, University of Gondar, Chechela street, Lideta subcity kebele 16, Gondar, Ethiopia

**Keywords:** Antidiarrheal, Indigofera spicata, Castor oil, Ethnopharmacology

## Abstract

**Background:**

Till now many of medicinal plants having claimed therapeutic value traditionally are waiting scientific verification of their efficacy and safety. Accordingly this study is conducted to evaluate the antidiarrheal activity of hydromethanolic root extract of *Indigofera spicata* Forssk. in castor oil induced diarrhea model, misoprostol induced secretion model and its antimotility activity using charcoal as a marker.

**Methods:**

In all the three models the animals were randomly allocated into five groups of six animals each and then group I mice were received 1 ml/100 g normal saline, group II were treated with standard drug as a positive control whereas group III, IV and V were treated with 100, 200 and 400 mg/kg extract doses, respectively. Statistical significance of differences in the mean of number of defecations, fluid content of faces, intestinal fluid accumulation ratio, intestinal fluid weight and distance travelled by charcoal between groups was analyzed by SPSS version-21 using one way ANOVA followed by Tukey’s post hoc multiple comparison.

**Result:**

The hydromethanolic crude extract of Indigofera spicata at 200 and 400 mg/kg mg/kg doses showed statistically significant (*p* < 0.05) inhibition of the frequency of defecation and weight difference of the fluid content of the faces compared to the negative controls. For those doses the percentage inhibition of diarrheal feces was 43.62 and 53.51 %, respectively. The antisecretary activity of the extract in terms of fluid accumulation ratio was not found significant but in terms of intestinal fluid weight, all the extract doses revealed significant (*p* < 0.05) inhibition. Unlike the standard drug, the antimotility activity of the extract was not found statistically significant compared to the negative control.

**Conclusion:**

Root of *Indigofera spicata* Forssk. has shown promising antidiarrheal activity which validates its traditional use. Further studies are needed and possibly the plant may serve as a potential source of new agent in the therapeutic armamentarium of diarrhea.

## Background

Diarrhea is generally defined as the passage of abnormally liquid or unformed stools associated with increased frequency of defecation, and abdominal pain [1]. Despite reductions in morbidity and mortality worldwide, diarrhea still accounts for more than 2 million deaths annually and is associated with impaired physical and cognitive development in resource-limited countries [2, 3]. This is more significant in case of infants and children for instance in developing countries, diarrhoeal disease accounts for an estimated 17.5–21 % of all deaths in children under the age of 5 years, equivalent to 1.5 million deaths per year. Of all child deaths from diarrhoea, 78 % occur in the African and South-East Asian regions, creating a tremendous economic strain on healthcare costs [4].

The existence of unique geographic, economic, political, sociocultural, and personal factors in sub-Saharan region interact to create distinctive continuing challenges to diarrhea prevention and control. Consequently, approximately 40 % of childhood deaths from diarrhea worldwide will occur in Sub-Saharan Africa by the year 2000, although only 19 % of the world’s population under the age of 5 years will live in this region. This continuing epidemic deserves sustained programmatic and research attention as international public health moves on to confront newer issues in infectious disease and the changing burdens of disease associated with the demographic transition [5, 6].

Medicinal plants are usually preferred to treat gastrointestinal disorders, for example, constipation and diarrhea, because they contain multiple constituents with effect-enhancing and/or side effect-neutralizing potential [7] and, hence are considered relatively safe in prolonged use. There has been increased global interest in traditional medicine and there are efforts underway to monitor and regulate herbal drugs and traditional medicine [8]. Due to reliance of the society of resource limited areas still on herbal medicine for their health care needs WHO recommended the integrated use of folk and modern medicine for controlling of health problems [9].

Like other developing countries, people in Ethiopia strongly rely on therapeutic benefits of traditional medicine. Accordingly, ethnobotanical survey studies reported that there are a number of plants which do have claimed antidiarheal role but scientific therapeutic and safety evaluations on some of these herbs such as *Indigofera spicata* Forssk. have not been reported [10–12].

Indigofera species posses multiple uses ranging from several economical and ecological roles, feed for livestock, ornamental, medicinal plant recipes as well as dye for commercial purpose. In this genus and family Fabaceae, the flowering herb *Indigofera spicata* Forssk. is traditionally useful for the treatment of meningitis, diarrhea, stomachache, diabetes and other health problems [10–16]. Apart from a number of ethnomedicine survey reports of this plant therapeutic usefulness for diarrhea and stomachache [10–12, 15], pharmacologically *Indigofera spicata* has reported in vitro antioxidant, cytotoxic and antibacterial [17–19] and in vivo antidiabetic activity [16]. It is stated that diarrhea impairs intestinal antioxidant defense system which will make it complicated and induce other oxidative stress disorders and therefore antioxidants may take up important application in the management of diarrhea [20–22]. Even though there is no detail study on the phytoconstituents of this plant previous works [16, 17] reported that this plant contains alkaloids, flavnoids, tannins, steroids and others most of which do have known antidiarrheal activity [23, 24]. Thus, considering all these the present study is aimed at pharmacologic verification of the antidiahreal activity of *Indigofera spicata* Forssk. using animal model that may give us a clue to enlighten on its efficacy for diarrhea and the essence of further investigations.

## Methods

### Plant material

The roots of *Indigofera spicata* (IS) were collected from Shawra and its surounding area, South West Gondar province, North West Ethiopia in December, 2014. Taxonomic identification was done by a botanist (Mr. Abiyu Enyew, Plant biology and biodiversity (PHD candidate), Addis Abeba Univesity) and a voucher specimen (EM001) is already deposited in the Department of Biology, National Herbarium, Addis Ababa University.

### Experimental animals

Healthy male Swiss albino mice, weighing between 22–32 g and 6 to 8 weeks old, were obtained from animal house of Department of Pharmacology, University of Gondar. They were housed in plastic cages with softwood shavings and chips as beddings. They were maintained under standard condition of relative humidity, temperature and 12 h light /12 h dark cycle and given food and water *ad labtium*. All mice were acclimatized to the working environment 1 week before the beginning of pharmacologic activities evaluation [25, 26].

### Extraction procedure

The fresh roots of *Indigofera spicata* Forssk. were thoroughly washed with distilled water to remove dirt and soil, and dried under shade and optimal ventilation for 2 weeks. The dried roots were further chopped into small pieces and reduced to powder using electronic miller. The coarsely powdered roots were subjected to maceration extraction procedure using 80 % methanol (for 72 h at room temperature) as a menstruum and this was done three times. The respective extract was filtered using Whatman No-1 filter paper and the solvent was evaporated in an oven under reduced pressure at 40 °C. Finally the dried extract was stored at 4 °C in refrigerator.

### Prelimnary phytochemical screening

The presence and absence of secondary plant metabolites in the crude hydromethanolic root extract of *Indigofera spicata* was screened by color forming and precipitation assays using standard procedures [27, 28].

### Acute toxicity

As per the OECD guideline, acute oral toxicity was determined by using the limit dose of 2000 mg/kg body weight of the mice [26].

### Animal grouping and dosing

In all models the mice were randomly grouped (*n* = 6) and the substances administered were as follows:

Group I: received 1 ml/100 mg normal saline (NS).
Group II: treated with standard drug, 3 mg/kg lopiramide (3 mg/kg Lop) in both castor oil induced antidiahreal test and misoprostol induced antisecretory test and atropine sulfate 0.1ug/g IP for gastrointestinal motility test.
Group III: treated with 100 mg/kg extract (100 mg/kg ISP).
Group IV: treated with 200 mg/kg extract (200 mg/kg ISP).
Group V: treated with 400 mg/kg extract (400 mg/kg ISP).

The test doses of the extract were selected based on the acute toxicity study result. Volume to be administered was also determined based on OECD guideline which states 1 ml/100 g of body weight of the animal [26]. As people traditionally use the preparations of the plant extract via oral route using water as a vehicle [9, 10] the study was conducted using oral route of administration except the intraperitoneal injection of atropine, standard drug.

### Antidiarrheal activity evaluation of 80 % methanolic root extract of *I. spicata*

In doing this, three antidiarrheal activity evaluation models were used. The first was the castor oil induced diarrheal model which is helpful to evaluate the overall possible antidiarrheal activity of the plant material. And this was followed by the attempt of investigating the antidiarrheal mechanism of action of the plant extract i.e. either by inhibition of intestinal transit and/or antisecretary activity.

#### Castor oil induced model

The assimilation of methods described by Shoba [29] and Franca [30] with modifications were followed for this study. Only animals which were found diarrheic when they have taken 0.5 ml castor oil in the initial screening test were included in this experiment. Mice fasted for 24 h were randomly allocated to five groups of six animals each. For the induction of diarrhea 0.5 ml castor oil was given for each mouse 30 min before treatment. Each animal was then placed in individual cage, the floor of which was lined with transparent paper and every hour the floor lining was changed. Onsets of diarrhea, total number of fecal outputs within in the 4 h period were recorded. And even total fluid content of the faces was determined by using the weight difference of the fresh and dry stool (dried for 24 h at room temperature in a shaded area). Evacuation classification based on stool consistency was assigned as follows: normal stool = 1, semi-solid stool = 2 and watery stool = 3 and mean of evacuation index (EI) was calculated for each group [31]. For all the groups the percentage inhibition of diarrhea was also calculated compared to the negative controls.

#### Gastrointestinal motility test by charcoal meal

The mice were first fasted for 18 h, but had free access to water. After the grouping, the respective groups were treated as mentioned above. After 1 h each animal was loaded with 1 ml of 3 % deactivated charcoal in normal saline and then waited for 1 h and dissected. The small intestine (from pylorus to caecum) was removed and its length was measured. The intestinal charcoal transit was expressed as a percentage of the distance moved by charcoal to the total length between the pylorus and the caecum [32]. The known spasmolytic agent, atropine was used as a standard drug for positive control group.

#### Antisecretary assay

Prostaglandins (PGE_2_) induce intestinal secretion and this is helpful to evaluate antisecretary activity of different chemical compounds [33, 34]. Thus, in this model the mice were first fasted for 24 h and then received 20ug/kg misoprostol for the induction of intestinal secretion. After 1 h each group of mice were treated just like the above castor oil induced diarrhea model. The animals were then sacrificed after 24 h by cervical dislocation, laparotimized, and then the pyloric and caecal ends of the small intestine were tied and removed. For each animal fluid accumulation ratio (the weight of intestine to the rest of the body weight of mouse) was determined and the antisecretory activity was expressed in percentage of inhibition [35]. The weight of the fluid content of the removed intestine was also determined by subtracting the weight of the intestine before and after milking of the removed intestine.

### Statistical analysis

All the experimental data are expressed as mean ± Standard error of means. For data processing and analysis SPSS statistical software Version 21.0 was used. Statistical significance of differences between groups was assessed by One-way ANOVA followed by post-hoc Tukey’s multiple Comparison Test. The results were considered significant at *p*-value less than 0.05.

### Ethical clearance

All the experimental animals were handled and used according to the animal care and welfare guidelines [25, 26, 36]. The experimental protocols were requested to and approved by the Institutional Review Board of the College of Medicine and Health Sciences, University of Gondar and ethical clearance was obtained from the research and publication office of the University.

## Result

### Extract material

The final dried hydromethanolic root extract of *Indigofera spicata* was brown powder in its color and the yield was 12.53 %.

### Phytochemical screening result

The preliminary phytochemical screening test results on the selected secondary plant metabolites are summarized in Table 1.

**Table 1:** Preliminary phytochemical screening result of crude hydromethanolic root extract of *Indigofera spicata*

Test	Result
Alkaloids	+
Tannins	+
Saponins	+
glycosides	+
Flavonoids	+
Phenolic compounds	−
Steroidal compounds	+
Terpenoids	+

+ refers presence and − refers absence

### Oral acute toxicity

The oral acute toxicity test by using the limit dose of 2000 mg/kg body weight of the mouse found safe because at this dose the animals didn’t show any observable physical and behavioral changes, confirming that the LD50 of the extract is greater than 2000 mg/kg.

### Andiarrheal acitivity evaluations

#### Effect of the extract on the castor oil induced diarrhea

Considering the latency of defecation after castor oil supplementation, only the 400 mg/kg extract dose treated groups demonstrated significant (*p* < 0.01) delay compared to the negative controls. The onset of defecation in this group was also found significantly (*p* < 0.05) different compared to the 100 and 200 mg/kg extract dose treated groups. Like that of the standard drug, the hydromethanolic crude extract doses (200 and 400 mg/kg) of root of *Indigofera spicata* as it is sown in Table 2 showed statistically significant (*p* < 0.05) inhibition both in the frequency of defecation and total weight of the fluid content of the faces compared to the negative controls. The percentage inhibition of diarrhea by the 100, 200 and 400 mg/kg doses of the extract was determined 22.49, 43.62 and 53.51 %, respectively. And this inhibition, especially from the largest dose of extract was comparable with the inhibitory effect (51.02 %) of lopiramid. Neither the positive control nor the extract treated groups exhibited statistically significant difference in the mean evacuation index compared to the normal saline exposed groups. The mean evacuation index didn’t show apparent difference among any of the groups.

**Table 2:** Effect of crude hydromethanolic extract of root of *Indigofera spicata* on castor oil induced diarrhea in mice

Group	Onset of diarrhea (min)	Total number of faeces in 4 h (frequency of defecation in 4 h)	Mean evacuation index	Fluid content of the faces (g)	% Inhibition of diarrhea
NS	63.33 ± 5.58	11.83 ± 1.30	2.39 ± 0.09	0.78 ± 0.13	0.00
3 mg/kg Lop	99.17 ± 18.14	5.83 ± 0.75*	2.29 ± 0.15	0.36 ± 0.05*	51.02
100 mg/kg IS	71.83 ± 5.67	9.17 ± 0.70	2.34 ± 0.06	0.61 ± 0.11	22.49
200 mg/kg IS	75.33 ± 4.86	6.67 ± 1.52*	2.17 ± 0.08	0.32 ± 0.09*	43.62
400 mg/kg IS	121.17 ± 12.18*^ab^	5.50 ± 0.67*	1.96 ± 0.16	0.35 ± 0.08*	53.51

Values are mean ± S.E. (*n* = 6), * for *p* < 0.05 compared to the negative control, ^a^ for *p* < 0.05compared to 100 mg/kg IS and ^b^ for *p* < 0.05 compared to 200 mg/kg IS

#### Gastrointestinal motility test

Regarding spasmolytic activity, all the extract dose treated groups didn’t show significant difference compared to the negative control and amongst each other. But the atropine treated group showed statistically significant (*p* < 0.001) inhibition of intestinal motility compared to both the negative control and all the extract treated groups as it is illustrated in Table 3. Compared to the normal saline exposed group, the percentage inhibition of intestinal transit from atropine and the largest dose of the extract (400 mg/kg) were found 68.45 and 11.24 %, respectively.

**Table 3:** Effect of crude hydromethanolic root extract of root of *Indigofera spicata* on intestinal motility using charcoal as a marker in mice

Group	Total intestinal length (cm)	Distance travelled by charcoal (cm)	% Intestinal transit of charcoal	% Inhibition
NS	55.67 ± 2.16	49.67 ± 2.06^††^	89.23 ± 1.26^††^	0.00
Atropine	57.83 ± 1.14	16.33 ± 1.78	28.15 ± 2.90	68.45
100 mg/kg IS	54.17 ± 2.15	47.67 ± 1.45^††^	88.21 ± 1.62^††^	1.14
200 mg/kg IS	53.17 ± 1.54	45.17 ± 1.89^††^	85.13 ± 3.47^††^	4.59
400 mg/kg IS	58.33 ± 1.36	46.17 ± 1.94^††^	79.20 ± 3.11^††^	11.24

Values are mean ± S.E. (*n* = 6), ^††^ refers *p* < 0.001 compared to the positive control

#### Anti-secretary activity

The antisecretary activity of the extract on misoprostol induced GI secretion according to the fluid accumulation ratio unlike that of lopiramide was found statistically insignificant compared to the negative control. As it is shown in Table 4 the percentage inhibition of intestinal fluid accumulation ratio of the extract is inversely related to its dose. Nevertheless, the weight of the intestinal fluid in all the respective extract doses treated groups was found significantly (*p* < 0.01) lower than the negative control and this was comparable with the lopiramide treated group.

**Table 4:** Effect of crude hydromethanolic extract of root of *Indigofera spicata* on misoprostol induced GI secretion in mice

Group	Weight of small intestine (g)	Intestinal fluid accumulation ratio	Weight of intestinal fluid (g)	% Inhibition of intestinal fluid accumulation ratio
NS	1.94 ± 0.52	0.109 ± 0.008	0.4567 ± 0.11	0.00
3 mg/kg Lop	1.38 ± 0.81	0.084 ± 0.004*	0.1567 ± 0.01*	22.81
100 mg/kg IS	1.46 ± 0.35	0.088 ± 0.004	0.1367 ± 0.03*	19.21
200 mg/kg IS	1.45 ± 0.67	0.090 ± 0.006	0.1383 ± 0.01*	17.27
400 mg/kg IS	1.59 ± 0.41	0.096 ± 0.005	0.1650 ± 0.03*	11.81

Values are mean ± S.E. (*n* = 6), * for *p* < 0.05 compared to the negative control

## Discussion

In so many areas in Ethiopia *Indigofera spicata* is used for the treatment of different health problems including diarrhea [9–15] without scientific substantiation of its safety and efficacy. The safety issue is highly important because this plant has demonstrated hepatotoxic, abortifacient like and teratogenic effects in animals [37–39].

Diarrhea may occur when there is a change in active ion transport by decreased sodium absorption or increased chloride secretion, change in intestinal motility, increase in luminal osmolarity; and/or increase in tissue hydrostatic pressure [40]. From all these mechanisms castor oil via its active compound ricinoleic acid induces diarrhea by stimulating secretory processes and intestinal motility secondary to irritation and inflammation [41]. The antidiarrheal agents act by inhibiting one or more of the aforementioned pathophysiologic processes.

Basically in folk medicine root of *Indigofera spicata* is used for treatment of diarrhea by using water as a vehicle but here in this study we used 80 % methanol because hydromethanolic solvents (especially 80 % methanol) are usually better and more efficient in extracting the most important bioconstituents of the plant material which is owing to their expanded polarity range. By virtue of the cosolubility, many compounds, which are insoluble individually in pure state in methanol could be extracted quite easily with hydroalcoholic solvents [42–44].

Here in the oral acute toxicity test as per the 2000 mg/kg limit dose of the OECD guideline was performed to get a clue of the appropriate safe dose range that could be used in evaluating the antidiarrheal activity of the extract in different models instead of clarifying all the toxicity profile of the crude extract. Accordingly in agreement with the previous oral acute toxicity report of this plant leaves extract [16] and other species of genus *Indigofera* [45] the LD50 of crude hydromethanolic root extract of *Indigofera spicata* was found greater than 2000 mg/kg body weight of the animal.

As it shown in Table 2 in the castor oil induced diarrhea the root extract of *Indigofera spicata* inhibits frequency of defecation and fluid content of stool more likely in a dose dependent manner. For instance with in 4 h period of post castor oil exposure, the frequency of defecation and fluid content of the stool unlike the 100 mg/kg in those treated with 200 and 400 mg/kg extract doses there was statistically significant difference (i.e. inhibition) compared to the normal saline exposed mice. The percentage inhibition of frequency of defecation from the 400 mg/kg dose of the extract is 53.51 % which is nearly equivalent with 51.02 % of the positive control. This antidiarrheal activity of *Indigofera spicata* in a dose dependent manner makes it more similar with other plant extracts [46]. However, the mean evacuation index didn’t have statistically apparent difference among all groups which may be due to small sample size and/or methodologic limitation in designating evacuation based on stool consistency.

Contrasting to the antidiarrheal activity of the extract in the castor oil induced diarrhea model, its activity on intestinal motility was not found statistically significant (*p* > 0.05) and its is confirmed that the plant material don’t have essential antimotility activity. However, considering the percentage inhibition of intestinal transit as it is shown in Table 3 as the dose increased by two fold the response also increased by more than two fold and hence the extract still do have dose dependent activity in this model.

In the antisecretary activity evaluation only the intestinal fluid accumulation ratio of lopiramide treated group was found statistically significant compared to the negative controls. But in this model the weight of the intestinal fluid (i.e. determined by taking the weight difference of the dissected intestine before and after 24 h) like the positive control in all the extract dose treated groups shown significant difference (*p* < 0.05) compared to the normal saline exposed mice. Furthermore, the percentage inhibition of intestinal fluid accumulation ratio by 100, 200, and 400 mg/kg doses of the extract was determined to be 19.21, 17.27 and 11.81 % respectively. This tells us the presence of noticeable relationship between the dose and response of the extract. In this misoprostol induced intestinal secretion model the antisecretary activity of the plant demonstrated inversely associated dose–response relationship. To the contrary as it is shown in the castor oil induced model the antidiarrheal activity of the larger doses is better than the lower dose of the extract. This could be explained by difference in secretion induction mechanism of castor oil and misoprostol that may affect the antidiarrheal activity of the plant extract. Furthermore, considering lack of significant antimotility activity and antisecretary activity using intestinal fluid accumulation ratio as a parameter but not in terms of weight of intestinal fluid there may be a possibility of the plant extract to have antidiarrheal activities via adsorbent like mechanism of action. In conclusion, the antidiarrheal mechanism of action of the extract need to be investigated further since the overall finding of this study never address the definitive mechanism.

Regarding the preliminary phytochemical screening in agreement with the previous report on its leaves extract [16] and related species [45] in the crude hydromethanolic root extract of *Indigofera spicata* the test was found positive for alkaloids, flavonoids, tannins, glycosides, saponnins and some other secondary plant metabolites as it is depicted in Table 1. And these secondary plant metabolites have known antidiarrheal activity independently or synergistically [23].

Among plant phytochemicals it is reported that tannins reduce diarrhea by reducing intestinal secretion mediated by the denaturation of secretary proteins and inhibiting the motility of intestine via altering the intracellular Ca^+2^ level [24]. Likewise, flavnoids are also known intestinal motility and water and electrolyte secretion inhibitors and even they do have antioxidant activity which may have a role in suppressing the catalytic activity of different enzymes including enzymes for the synthesis of prostaglandins [22, 47]. Parallel to this fact it is reported that *Indigofera spicata* do have antioxidant and cytotoxic activity [17, 18] that may have a role in its antidiarrheal mechanism of action. Furthermore, steroids may also enhance the absorption of hydroelectrolytes across the intestinal lumen [23, 48]. Thus the demonstrated biologic antidiarreal activity of hydromethanolic crude root extract of *Indigofera spicata* might be due to the presence of the aforementioned secondary plant metabolites.

## Conclusion

In conclusion this study verified that other than being safe up to a dose of 2000 mg/kg, hydromethanolic crude root extract of *Indigofera spicata* Forssk. has antidiarrrheal activity. Accordingly, the study validates traditional use of the plant for diarrhea and may guide us to use it as a potential source of new agent in the therapeutic armamentarium of diarrhea.
